# Спорадический первичный гиперпаратиреоз с множественной трансформацией околощитовидных желез

**DOI:** 10.14341/probl12798

**Published:** 2021-12-30

**Authors:** Е. Е. Бибик, А. К. Еремкина, О. А. Князева, Н. Г. Мокрышева

**Affiliations:** Национальный медицинский исследовательский центр эндокринологии Минздрава России; Национальный медицинский исследовательский центр эндокринологии Минздрава России; Общество с ограниченной ответственностью «Альтамед+»; Национальный медицинский исследовательский центр эндокринологии Минздрава России

**Keywords:** первичный гиперпаратиреоз, гиперкальциемия, множественные аденомы, околощитовидные железы, синдром множественных эндокринных неоплазий, рак околощитовидных желез

## Abstract

Полигландулярное поражение околощитовидных желез (ОЩЖ) при первичном гиперпаратиреозе (ПГПТ) может быть спорадическим или развиваться в рамках наследственных синдромов, манифестируя в молодом возрасте. Представлено описание тяжелой спорадической формы ПГПТ с гигантскими новообразованиями ОЩЖ у молодой пациентки. Клинические данные позволяли заподозрить МЭН-1 синдром или карциномы ОЩЖ, однако мутации CDKN, CDC73, MEN1 были исключены. Пациентке выполнено удаление трех выявленных опухолей: доброкачественных аденом левых ОЩЖ и гиперплазии правой ОЩЖ. Послеоперационная гипокальциемия и тяжелый синдром голодных костей потребовали назначения препаратов витамина D, карбоната кальция. Однако через год после операции подтвержден «мягкий» рецидив заболевания. С учетом отказа пациентки от повторной операции и значительного улучшения состояния органов-мишеней продолжено активное наблюдение. Пациентка нуждается в дальнейшем тщательном динамическом контроле специалистами с целью своевременного выявления показаний к повторному хирургическому лечению для улучшения качества и увеличения продолжительности жизни.

## Abstract (en)

Multiple lesions of the parathyroid glands (PTG) in primary hyperparathyroidism (PHPT) can be sporadic or develop as part of hereditary syndromes, manifesting at young age. There the description of a severe sporadic PHPT with big parathyroid neoplasms in the young patient is presented. Clinical data made it possible to suspect MEN-1 syndrome or parathyroid carcinomas; however, mutations CDKN, CDC73, MEN1 were excluded. The patient underwent removal of three identified tumors: benign adenomas of the left PTG and hyperplasia of the right one. Postoperative hypocalcemia and severe hungry bone syndrome required the administration of vitamin D and calcium carbonate preparations. However, a year after the operation, a «mild» recurrent disease was confirmed. Taking into account the patient’s refusal to reoperation and a significant improvement of the target organs state, active observation was continued. The patient needs further careful dynamic monitoring by specialists in order to timely identify indications for repeated surgical treatment to improve the life quality and span.

## АКТУАЛЬНОСТЬ

Первичный гиперпаратиреоз (ПГПТ) является относительно распространенным эндокринным заболеванием, характеризующимся верхненормальным или повышенным уровнем кальция сыворотки крови вследствие избыточной продукции паратиреоидного гормона (ПТГ) патологически измененными околощитовидными железами (ОЩЖ). ПГПТ в 80–85% случаев обусловлен солитарной аденомой, в 10–15% случаев — гиперплазией или множественными аденомами нескольких или всех ОЩЖ (полигландулярное поражение), в 1–5% — карциномой ОЩЖ [1]. Примерно в 5–10% случаев ПГПТ развивается в рамках генетически детерминированных синдромов, чаще в составе множественной эндокринной неоплазии 1 типа (МЭН-1), реже — множественной эндокринной неоплазии 2 и 4 типов (МЭН-2, МЭН-4), семейного изолированного гиперпаратиреоза и синдрома гиперпаратиреоза с опухолью нижней челюсти (HPT-JT) [1]. Несмотря на то что для наследственной формы ПГПТ, ассоциированной с герминальными мутациями, характерны синхронные или метахронные патологические изменения ОЩЖ, в клинической практике случаи полигландулярного поражения чаще носят спорадический характер. По данным литературы, распространенность множественного поражения ОЩЖ при спорадическом ПГПТ варьирует от 7 до 33% наблюдений. В представленных исследованиях в морфологической структуре преобладают гиперплазии нескольких или всех ОЩЖ, реже регистрируются аденомы двух или крайне редко — трех желез [2][3].

ПГПТ встречается во всех возрастных группах, в том числе у детей и подростков, однако наибольшую распространенность он имеет у женщин в постменопаузальном периоде. Развитие ПГПТ у лиц молодого возраста подозрительно в отношении наследственных форм заболевания, что требует расширенного лабораторно-инструментального обследования для своевременной постановки диагноза. В настоящий момент открыт вопрос о необходимости рутинного молекулярно-генетического скрининга среди всех молодых пациентов с ПГПТ [4][5]. Вероятность выявить специфическую генетическую причину ПГПТ выше при наименьшем возрасте дебюта заболевания, однако рекомендаций о генетическом скрининге в возрасте до 30, 35 или 40 лет не сформулировано [6]. Тем не менее отдельно можно выделить группу пациентов с ПГПТ моложе 40 лет с сочетанным полигландулярным поражением ОЩЖ как наиболее подозрительную в отношении синдрома МЭН-1, даже несмотря на отсутствие отягощенного семейного анамнеза. Результаты пилотных исследований российской популяции свидетельствуют о смещении возраста манифестации заболевания на 3-ю декаду жизни, в связи с чем рекомендуемый возрастной порог для проведения генетического исследования составляет 40 лет [1].

Мы представляем случай рецидивирующего течения ПГПТ у молодой пациентки с множественным поражением ОЩЖ в отсутствие мутаций генов, ассоциированных с наследственными причинами заболевания.

## ОПИСАНИЕ СЛУЧАЯ

Пациентка С., 34 лет, впервые стала отмечать постоянные боли и слабость в ногах после перенесенного перелома основной фаланги V пальца правой стопы в 2017 г. При рентгеновской денситометрии было выявлено снижение показателя минеральной плотности кости (МПК) в поясничном отделе позвоночника (L1–L4) до -2,6 SD по Z-критерию. Кроме того, обнаружены повышение концентрации паратиреоидного гормона (ПТГ) до 242 пг/мл и недостаточность витамина D (26 нг/мл), уровень кальциемии не определялся. Пациентке назначена терапия альфакальцидолом в дозе 1 мкг/сут, однако в связи с развитием почечной колики через 1 мес лечения препарат был отменен. В последующем отмечалось прогрессирующее ухудшение самочувствия пациентки в виде нарастания общей слабости, интенсивных болей в спине и нижних конечностях, разрушения зубов. При дополнительном обследовании впервые выявлена гиперкальциемия (кальций ионизированный 1,62 ммоль/л) и диагностирован ПГПТ. С целью топической диагностики по месту жительства выполнена планарная сцинтиграфия, по результатам которой зафиксировано два очага гиперфиксации радиофармпрепарата (РФП) с тенденцией к слиянию на уровне нижнего полюса левой доли щитовидной железы.

Осенью 2018 г. (спустя год после первых проявлений заболевания) пациентка госпитализирована в отделение патологии ОЩЖ ФГБУ «НМИЦ эндокринологии» Минздрава России с вышеуказанными жалобами.

Результаты физикального, лабораторного и инструментального исследования

При осмотре отмечено нормостеническое телосложение (вес 72 кг, рост 172 см, индекс массы тела 24,3 кг/м2), обращали на себя внимание костный выступ в проекции грудинно-ключичного сочленения справа, деструкция и деформация зубов, ярко выраженный тревожный синдром с высоко-нормальными показателями гемодинамики (ЧСС 80 в минуту, АД 135–150/80–90 мм рт. ст.). При лабораторном обследовании зафиксированы значимое повышение концентрации кальция крови и ПТГ, гиперкальциурия. С целью коррекции гиперкальциемии был назначен цинакальцет с титрацией дозы до 60 мг в сутки, регидратационная терапия, однако значимого эффекта не наблюдалось: сохранялось значимое повышение уровня альбумин-скорректированного кальция крови (табл. 1). Тем не менее для профилактики прогрессирования гиперкальциемии было принято решение о продолжении терапии цинакальцетом 30 мг в сутки.

По данным рентгеновской денситометрии подтверждено снижение МПК относительно возраста до уровня остеопороза во всех отделах скелета (табл. 2). При рентгенографии выявлены начальная компрессия L1-позвонка (5% потери высоты) и компрессионный перелом Th12-позвонка (22% потери высоты), уплотнение стенок брюшного и грудного отделов аорты, при мультиспиральной компьютерной томографии (МСКТ) — микролиты в обеих почках, а также образования мягкотканной плотности на уровне рукоятки и тела грудины до 11,2×11,7×10,7 мм, с четкими, склерозированными контурами и неравномерным истончением кортикального слоя, что расценено как «бурые» опухоли — проявления фиброзно-кистозного остеита.

В рамках первого этапа топической диагностики проведено ультразвуковое исследование (УЗИ). Слева за нижней третью щитовидной железы визуализировано образование пониженной эхогенности с ровными контурами 7,2×2,6×1,3 см, распространяющееся вниз и загрудинно, до дуги аорты, охватывающее общую сонную артерию. С учетом крупных размеров опухоли, значимых изменений лабораторных показателей и тяжести течения заболевания, нельзя было исключить злокачественный характер патологии. В качестве дополнительных инструментальных методов проведены сцинтиграфия с однофотонной эмиссионной компьютерной томографией (ОФЭКТ/КТ), МСКТ шеи с контрастным усилением и диффузионная магнитно-резонансная томография (МРТ). В результате выявлены 3 образования ОЩЖ, два из которых более 3 см в диаметре: левая верхняя 1,5×1,2×3,9 см, атипично расположенная левая нижняя 2,7×2,2×5,9 см; правая нижняя 0,7×0,4×1,6 см (рис. 1–3).

Принимая во внимание молодой возраст пациентки и множественное поражение ОЩЖ, выполнен комплексный лабораторно-инструментальный скрининг классических компонентов МЭН-1 синдрома. Показатели адренокортикотропного гормона (утром 23,05 пг/мл, вечером 7,64 пг/мл), инсулиноподобного фактора роста-1 (135,8 нг/мл), пролактина (193,4 мЕд/л), тиреотропного гормона (1,7 мМЕ/л) сохранялись в пределах референсных значений. Впервые были зафиксированы лабораторные признаки эндогенного гиперкортицизма (гиперкортизолемия на фоне сохранения ритма секреции кортизола (утром 723,5 нмоль/л, вечером 307,3 нмоль/л), повышение свободного кортизола в моче до 1220,6 нмоль/сут, в ходе ночного подавляющего теста с 1 мг дексаметазона кортизол крови утром 72,61 нмоль/л). Кроме того, выявлено незначительное повышение концентраций метанефрина (335,75 мкг/сут) и норметанефрина (458,49 мкг/сут) в суточной моче. По данным МСКТ объемных образований в проекции поджелудочной железы не визуализировалось, наблюдалась сегментарная гиперплазия обоих надпочечников. При отсутствии явных клинических признаков гиперкортицизма, изменений показателей адренокортикотропного гормона и значимой психоэмоциональной лабильности пациентки на фоне выраженной гиперкальциемии отклонения гормональных показателей расценены как функциональный гиперкортицизм вследствие болевого синдрома, развивающегося тревожно-депрессивного расстройства. Рекомендовано исследование основных параметров стероидного обмена в динамике после достижения ремиссии ПГПТ.

Проводилось обследование кровных родственников пациентки. Данных за патологические изменения фосфорно-кальциевого обмена не получено (табл. 3).

**Table:** Таблица 1. Основные биохимические и гормональные показатели пациентки С.

Показатель/Дата	Референсные значения	2018 г.	2019 г.	2020 г.
при поступлении	через 6 сут	через 11 сут	ранний п/опер период	при поступлении	через 7 сут
Кальций ион.,ммоль/л	1,03–1,29	1,38	1,42	1,41	1,14	-	-
Альбумин-скоррект. кальций, ммоль/л	2,15–2,55	3	3,1	3	-	2,4	2,57
ПТГ, пг/мл	16–65	403,7	466	-	57,11	80,25	113
Фосфор, ммоль/л	0,74–1,52	0,53	-	-	-	0,81	-
СКФ (EPI), мл/мин/1,73 м2		120	-	122	-	115	-
Кальций в суточной моче, ммоль/сут	2,5–8,0	-	10,9	-	-	6,57	-

**Table:** Таблица 2. Показатели рентгеновской денситометрии при динамическом наблюдении пациентки С.

Отдел скелета, Z-критерий/дата	2018 г.	2020 г.	Динамика МПК
L1–L4, SD	-2,8	-2,7	+8,3%
L4, SD	-3,4	-2,9	
Femur Neck, SD	-1,9	-1,8	+3,5%
Total Hip, SD	-1,5	-1,3	
Radius 33%, SD	-4,2	-4,6	-10%
Radius Total, SD	-4,9	-5,5	

**Figure:**
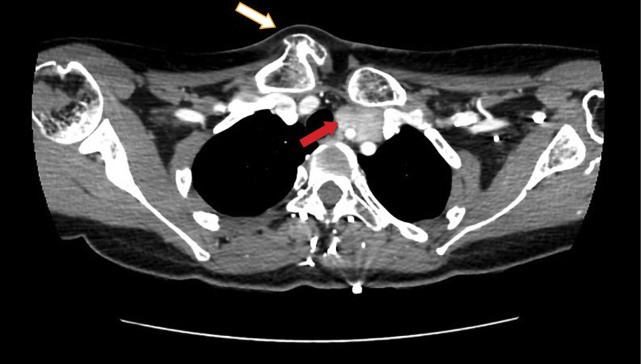
Рисунок 1. Мультиспиральная компьютерная томография шеи и верхнего средостения пациентки С. с контрастированием (белой стрелкой указана деформация правой ключицы, красной стрелкой — образование левой околощитовидной железы).

**Figure:**
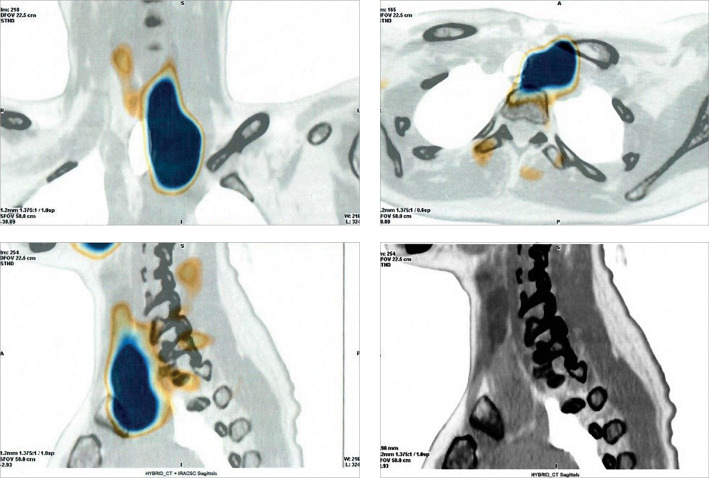
Рисунок 2. Однофотонная эмиссионная компьютерная томография пациентки С. Позади нижней трети правой доли щитовидной железы обнаружена овоидная структура с четкими ровными контурами 4×6×15 мм, низкоинтенсивно накапливающая радиофармпрепарат (РФП). Кзади и книзу от левой доли, латеральнее пищевода, определяется овоидная структура с нечеткими, ровными контурами 8×10×38 мм, накапливающая РФП. Латеральнее нее визуализирована крупная мягкотканная структура с четкими ровными контурами 22×30×53 мм, распространяющаяся от нижнего полюса левой доли книзу до уровня дуги аорты, накапливающая РФП.

**Figure:**
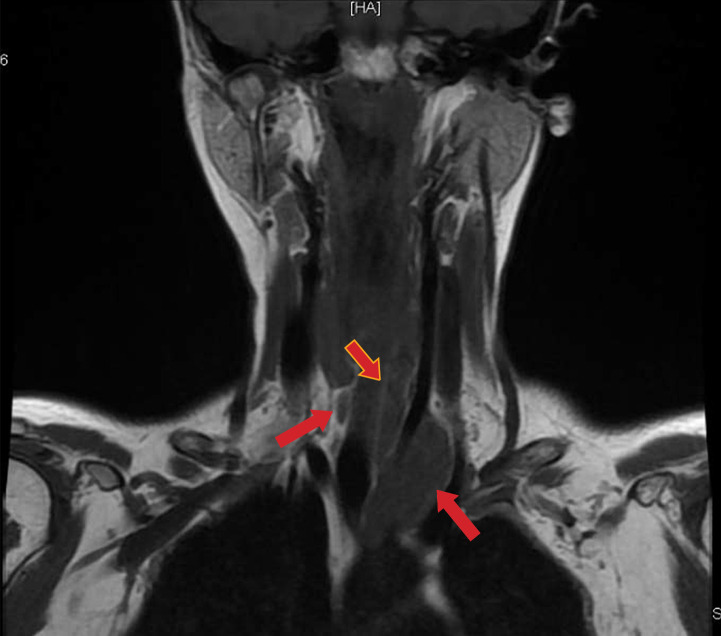
Рисунок 3. Диффузионная магнитно-резонансная томография шеи (красными стрелками указаны образования правой и левых околощитовидных желез).

**Table:** Таблица 3. Показатели минерального обмена у кровных родственников пациентки С.

Показатели	Референсные значения	Отец	Мать	Сестра 1	Сестра 2
Альбумин-скоррект. кальций, ммоль/л	2,20–2,55	2,17	2,3	2,16	2,24
ПТГ, пмоль/л	1,6–6,9	5,68	4,84	4,71	3,07

В связи с выраженной гиперкальциемией и высокой вероятностью развития гиперкальциемического криза пациентка направлена на хирургическое лечение. Однако паратиреоидэктомия была отсрочена в связи с неоднократными обострениями хронического тонзиллита и острыми инфекциями верхних дыхательных путей, требующими антибактериальной и дезинтоксикационной терапии. Пациентка оставалась на поддерживающей дозе цинакальцета 30 мг/сут. Весной 2019 г. выполнено хирургическое удаление правой нижней, левой верхней и атипично расположенной левой нижней ОЩЖ. Патоморфологическая картина соответствовала аденомам левых верхней и нижней ОЩЖ фолликулярного строения из главных клеток; правая нижняя ОЩЖ характеризовалась липоматозом и наличием участков фолликулярного строения.

В раннем послеоперационном периоде отмечена нормализация показателей кальция и ПТГ крови, назначена терапия альфакальцидолом 1,0 мкг/сут и колекальциферолом 25 000 МЕ/нед. Спустя 1 мес после хирургической операции при ухудшении общего самочувствия у пациентки выявлена значимая гипокальциемия на фоне дефицита витамина D (15,48 нг/мл), в связи с чем рекомендовано дополнительное лечение препаратами кальция 1000 мг/сут, насыщающей дозой колекальциферола (50 000 МЕ/нед в течение 2 мес с последующим переходом на поддерживающую дозу 15 000 МЕ/нед) с достижением нормокальциемии (табл. 1). Клинические признаки выраженного синдрома «голодных костей» наблюдались на протяжении года. По техническим причинам молекулярно-генетическое исследование проведено в отсроченном периоде, мутаций в генах MEN1, RET, CDKN1B, CDC73 не выявлено.

При последующей госпитализации в стационар в 2020 г. на фоне отмены альфакальцидола и препаратов кальция за 1 мес до госпитализации, продолжения приема колекальциферола в дозе 2000 МЕ/сут зафиксировано умеренное повышение ПТГ на фоне нормокальциемии, нормокальциурии (табл. 1). С целью дифференциальной диагностики гиперпаратиреоза проведена функциональная проба с альфакальцидолом 1 мкг/сут, по результатам которой через 7 дней выявлена умеренная гиперкальциемия с повышением уровня ПТГ до 113 пг/мл, свидетельствующая в пользу первичного поражения ОЩЖ и рецидива заболевания. При УЗИ обнаружено образование атипично расположенной ОЩЖ 1,8×1,1 см слева, в верхней части переднего средостения, подтвержденное результатами повторных МСКТ и сцинтиграфии с ОФЭКТ/КТ в 2020 г. Опухоль располагалась между брахицефальным стволом и левой общей сонной артерией, прилегая к левому контуру трахеи и переднему контуру пищевода. При оценке состояния органов-мишеней подтвержден двусторонний микролитиаз без динамики от предыдущего исследования (справа конкременты до 0,5 см, слева — до 0,4 см). По результатам рентгенденситометрии отмечена значимая положительная динамика МПК в позвоночнике и бедренной кости при резко выраженном снижении МПК лучевой кости (табл. 2). При рентгенографии подтвержден компрессионный перелом Th12 (30% потери массы), впервые выявлен перелом L5 (25% потери массы), более вероятно произошедший в дооперационный период при невозможности своевременного проведения паратиреоидэктомии. Принимая во внимание гиперкортицизм в анамнезе, проведено повторное исследование стероидного профиля, отклонений не выявлено (кортизол вечерней слюны 2,2 нмоль/л, кортизол крови утром на фоне ночного подавляющего теста с 1 мг дексаметазона 37,2 нмоль/л). С учетом отказа пациентки от повторного хирургического вмешательства, положительной динамики состояния основных органов-мишеней, нормокальциемии и умеренного повышения ПТГ, принято решение продолжить консервативную терапию колекальциферолом (2000 МЕ/сут) под динамическим контролем лабораторных показателей минерального обмена (1 раз в 3–6 мес), общего состояния с рассмотрением хирургической реоперации в случае ухудшения самочувствия и прогрессирования осложнений заболевания.

## ОБСУЖДЕНИЕ

Представленный клинический случай описывает изолированный ПГПТ с множественным поражением ОЩЖ у молодой пациентки. При характерном сочетании клинических признаков наследственных эндокринных синдромов в первую очередь был проведен диагностический скрининг компонентов синдрома МЭН-1 [7]. Комплексное лабораторно-инструментальное обследование позволило исключить у женщины патологии гипофиза, поджелудочной железы, надпочечников. Дополнительный скрининг нарушений фосфорно-кальциевого обмена у кровных родственников первой линии, а также результаты молекулярно-генетического анализа самой пациентки исключили известные наследственные варианты ПГПТ. Несколько похожих случаев пациентов молодого и среднего возраста было описано ранее в литературе [3][8].

По результатам зарубежных работ, каждый пятый больной ПГПТ может иметь несколько образований ОЩЖ. Основным патоморфологическим субстратом такой формы патологии является гиперплазия всех или 2–3 желез, развивающаяся синхронно или асинхронно. Также встречаются множественные аденомы ОЩЖ, которые, в отличие от солитарных, более вероятно являются поликлональными и представляют собой разные заболевания [2]. В тоже время в крупном американском исследовании с включением 384 пациентов со спорадическим ПГПТ у 7% были обнаружены аденомы двух ОЩЖ, причем преимущественное поражение обеих верхних желез (p=0,008), возможно, свидетельствует о первоначальной гиперплазии ОЩЖ, происходящих из четвертого жаберного кармана, а не об отдельных неопластических трансформациях [9]. Особенностью нашей пациентки С., согласно результатам послеоперационного гистологического исследования, является наличие двух крупных аденом слева в сочетании с гиперплазией третьей ОЩЖ с противоположной стороны.

Поиск как лабораторно-инструментальных, так и клинических предикторов множественного поражения ОЩЖ до настоящего времени не дал однозначных результатов. Комбинированная топическая диагностика может способствовать дооперационному выявлению всех образований или навести на мысль о множественном поражении. Так, отрицательные или дискордантные результаты визуализирующих методов имеют высокую прогностическую ценность в отношении полигландулярной формы заболевания [10]. Интраоперационный мониторинг ПТГ в 20–45% случаев не позволяет обнаружить множественные опухоли и дать объективную оценку радикальности хирургического лечения [11]. Выявленное на предоперационном этапе поражение нескольких ОЩЖ является показанием к двусторонней ревизии шеи. Некоторые хирурги в качестве маркера гиперфункции используют размеры ОЩЖ, что основано на успешной нормализации параметров и низкой частоте рецидивов, достигнутых при удалении всех визуально увеличенных желез во время операции [12]. Полагают, если диагноз ПГПТ не вызывает сомнений, персистирующее или рецидивирующее течение более вероятно связано с гиперплазиями ОЩЖ [2]. В нашем примере расширенная топическая диагностика позволила на дооперационном этапе определить локализацию патологических образований, однако труднодоступность расположения одного из них (близкое прилегание крупных артерий) препятствовала радикальному лечению заболевания.

Помимо множественного поражения ОЩЖ, для пациентки С. были характерны крупные размеры образований (до 4–6 см слева, до 1,6 см справа в наибольшем измерении) и тяжесть течения заболевания (рефрактерная к консервативной терапии гиперкальциемия, выраженное повышение ПТГ, множественные костно-висцеральные осложнения), что не исключало злокачественный характер патологии. Предоперационная диагностика карцином ОЩЖ затруднена из-за отсутствия специфических клинических и лабораторных маркеров, за исключением случаев с первоначальным выявлением отдаленных метастазов или появлением охриплости голоса в результате инвазивного роста опухоли с поражением гортанного нерва [13]. Перспективным, но труднодоступным методом является определение мутаций в гене CDC73, которые присутствуют у более чем 75% больных со злокачественными образованиями. Свыше 30% таких пациентов имеют герминативную мутацию CDC73 с высоким риском развития ПГПТ с опухолью челюсти [14]. Гистологический диагноз карциномы ОЩЖ также представляет собой сложную задачу, особенно в случаях повреждения капсулы образования ОЩЖ, и, кроме того, зависит от опыта врача-морфолога [15]. Злокачественная патология чаще устанавливается ретроспективно при возникновении рецидива первичной опухоли или появлении метастазов. Предоперационный диагноз карциномы ОЩЖ, основанный на сочетании клинико-лабораторных особенностей и интраоперационной картине, в ряде случаев позволяет провести радикальное хирургическое лечение [13][15]. Аналогично нашему, недавно было опубликовано описание пожилой пациентки с большим пальпируемым образованием ОЩЖ (5,0×2,5×2,5 см), подозрительным в отношении злокачественного поражения. У женщины наблюдалась значимая гиперкальциемия (кальций ионизированный 1,45 ммоль/л) при повышении ПТГ до 1629 пг/мл, однако при патоморфологическом исследовании подтверждена аденома ОЩЖ [16]. В свою очередь, японскими специалистами продемонстрирован уникальный случай тяжелого ПГПТ (кальций крови составил 14,8 мг/дл, или 3,69 ммоль/л, а ПТГ — 1039 пг/мл) с множественным поражением ОЩЖ, при котором одно из образований представляло собой карциному [17].

У пациентов с выраженными костными осложнениями ПГПТ в послеоперационном периоде, как правило, наблюдается гипокальциемия, требующая своевременного назначения препаратов кальция и активного метаболита витамина D [7]. Так называемый «синдром голодных костей» у нашей пациентки с фиброзно-кистозным остеитом на фоне диффузного снижения МПК сопровождался снижением концентрации сывороточного кальция до 1,67 ммоль/л вследствие его усиленного поглощения костной тканью и оссалгиями на протяжении длительного времени. Несмотря на это, через год после хирургического лечения у пациентки С. отмечена значимая положительная динамика МПК в осевых отделах скелета. По данным различных источников, прирост МПК за первые 1–4 года после успешной паратиреоидэктомии составляет от 6 до 84% [18]. Продолжительность консервативной терапии препаратами кальция и витамина D определяется клинической симптоматикой и тяжестью поражения скелета и часто достигает 12 мес и более [19]. С учетом нерадикально проведенного хирургического лечения и подтвержденного наличия остаточной ткани аденомы ОЩЖ наша пациентка через год прекратила прием карбоната кальция и альфакальцидола и продолжает лечение колекальциферолом под динамическим наблюдением эндокринолога. Такие пациенты нуждаются в регулярном лабораторном (не реже 1 раза в 3–6 мес) и инструментальном (не реже 1 раза в 6–12 мес) контроле состояния органов-мишеней (УЗИ почек, определение СКФ, рентгеновская денситометрия осевых отделов скелета и лучевой кости) с целью своевременного выявления показаний к повторному хирургическому лечению.

## ЗАКЛЮЧЕНИЕ

Спорадический ПГПТ с полигландулярным поражением у лиц молодого возраста встречается редко, что требует проведения расширенного лабораторно-инструментального обследования, в том числе скрининга других возможных компонентов наследственных МЭН-синдромов. Крупные аденомы ОЩЖ могут имитировать карциномы по тяжести течения ПГПТ и дооперационным морфологическим характеристикам образований. Полноценная предоперационная топическая диагностика с применением нескольких визуализирующих методов способствует проведению радикального хирургического лечения, однако персистирующее течение и рецидивы заболевания нередки. Такие пациенты нуждаются в дальнейшем тщательном динамическом контроле специалистами с целью своевременного выявления показаний к повторному хирургическому лечению для улучшения качества и увеличения продолжительности жизни.

## ДОПОЛНИТЕЛЬНАЯ ИНФОРМАЦИЯ

Источники финансирования. Исследование проведено при финансовой поддержке Министерства здравоохранения Российской Федерации в рамках выполнения государственного задания «Оптимизация Российского электронного реестра пациентов с первичным гиперпаратиреозом» № НИОКТР 121030100032-7.

Конфликт интересов. Авторы декларируют отсутствие явных и потенциальных конфликтов интересов, связанных с содержанием настоящей статьи.

Участие авторов. Еремкина А.К. — анализ клинических и литературных данных, написание и редактирование статьи; Бибик Е.Е. — получение, анализ и интерпретация клинических данных, написание и редактирование статьи; Князева О.А. — написание статьи, анализ литературных данных; Мокрышева Н.Г. — анализ клинических данных, редактирование статьи. Все авторы одобрили финальную версию статьи перед публикацией, выразили согласие нести ответственность за все аспекты работы, подразумевающую надлежащее изучение и решение вопросов, связанных с точностью или добросовестностью любой части работы.

Согласие пациента. Пациент добровольно подписал информированное согласие на публикацию персональной медицинской информации в обезличенной форме в данном журнале.
